# Author contributions to ecological publications: What does it mean to be an author in modern ecological research?

**DOI:** 10.1371/journal.pone.0179956

**Published:** 2017-06-26

**Authors:** John M. Logan, Sarah B. Bean, Andrew E. Myers

**Affiliations:** 1 Massachusetts Division of Marine Fisheries, New Bedford, Massachusetts, United States of America; 2 Buttonwood Park Zoo, New Bedford, Massachusetts, United States of America; 3 The Derryfield School, Manchester, New Hampshire, United States of America; Institut Català de Paleoecologia Humana i Evolució Social (IPHES), SPAIN

## Abstract

Authorship is a central element of scientific research carrying a variety of rewards and responsibilities, and while various guidelines exist, actual author contributions are often ambiguous. Inconsistent or limited contributions threaten to devalue authorship as intellectual currency and diminish authors’ responsibility for published content. Researchers have assessed author contributions in the medical literature and other research fields, but similar data for the field of ecological research are lacking. Authorship practices in ecological research are broadly representative of a variety of fields due to the cross-disciplinary nature of collaborations in ecological studies. To better understand author contributions to current research, we distributed a survey regarding co-author contributions to a random selection of 996 lead authors of manuscripts published in ecological journals in 2010. We obtained useable responses from 45% of surveyed authors. Reported lead author contributions in ecological research studies consistently included conception of the project idea, data collection, analysis, and writing. Middle and last author contributions instead showed a high level of individual variability. Lead authorship in ecology is well defined while secondary authorship is more ambiguous. Nearly half (48%) of all studies included in our survey had some level of non-compliance with Ecological Society of America (ESA) authorship guidelines and the majority of studies (78%) contained at least one co-author that did not meet International Committee of Medical Journal Editors (ICMJE) requirements. Incidence of non-compliance varied with lead author occupation and author position. The probability of a study including an author that was non-compliant with ESA guidelines was lowest for professor-led studies and highest for graduate student and post doctoral researcher-led studies. Among studies with > two co-authors, all lead authors met ESA guidelines and only 2% failed to meet ICMJE requirements. Middle (24% ESA, 63% ICMJE) and last (37% ESA, 60% ICMJE) authors had higher rates of non-compliance. The probability of a study containing a co-author that did not meet ESA or ICMJE requirements increased significantly with the number of co-authors per study although even studies with only two co-authors had a high probability of non-compliance of approximately 60% (ICMJE) and 15 to 40% (ESA). Given the variable and often limited contributions of authors in our survey and past studies of other research disciplines, institutions, journals, and scientific societies need to implement new approaches to instill meaning in authorship status. A byline approach may not alter author contributions but would better define individual contributions and reduce existing ambiguity regarding the meaning of authorship in modern ecological research.

## Introduction

Authorship is central to scientific research for both philosophical and pragmatic reasons [1], but consensus within the scientific community on its definition remains elusive [2]. Authorship of peer-reviewed literature can provide a personal sense of achievement and contribution to scientific progress, and an author’s publication record acts as a form of intellectual currency for academic appointments, promotion, and funding [1]. Authorship is part of a cycle of credit in which a researcher’s publication record defines her or his total value. This value is based partly on the number of articles published, prestige of journals where articles are published, names and reputations of co-authors, and article subject matter. All of these facets can influence career development by contributing to decisions of tenure and other appointments, grant awards, and collaborations [3]. In addition to these various credits, authorship also includes an element of responsibility [4]. Authors need to be able to defend their findings and the integrity of their work [1]. The publication process does not include a universal set of requirements to define what it means to be an author and opinions vary among individual researchers (e.g., [5,6]).

Many individual journals and scientific societies have put forth guidelines on authorship requirements [7–10]. The Ecological Society of America (ESA) states that authorship should only be awarded to researchers that conceived the ideas or experimental design, participated actively in the execution of the study, analyzed and interpreted the data, or wrote the manuscript [11]. Similar single-faceted criteria are required by the National Institute of Health and American Physical Society [12,13]. The International Committee of Medical Journal Editors (ICMJE) outlines similar criteria, but requires that authors perform multiple tasks. In addition to contributing to conception and design, acquisition of data, or data analysis and interpretation, the ICMJE guidelines also require that authors provide intellectual contributions to the writing process and provide final approval prior to publication [14]. Many organizations have guidelines modeled after the ICMJE guidelines (e.g., British Sociological Association [15]). Over 3,000 journals directly subscribe to the ICMJE guidelines [16], including many ecological journals (e.g., BMC Ecology, Frontiers in Ecology and Evolution, Fisheries Management and Ecology [17–19]). Dickson et al. (1978) proposed an intermediate set of requirements in which all co-authors should contribute significantly to writing as well as conception, design, data collection, and/or analysis [20]. Most journals with authorship guidelines require both research and writing contributions [10].

Bennett and Taylor (2003) [1] describe various “authorship irregularities” in scientific publications that are inconsistent with journal and society guidelines. Guest (also referred to as gift, honorary, or enforced) authorship describes individuals receiving authorship without meeting the necessary criteria put forth by the relevant journal or scientific society [1,2,21–25]. Pressured authorship is a specific form of guest authorship in which the undeserving author, or white bull *sensu* Kwok (2005) [26], has a higher status than the lead author [1]. A literature review of authorship practice studies identified power issues in authorship as a recurring problem for students in their interactions with professors and supervisors [27]. Ghost authorship refers to the opposite situation in which a legitimate author is not included as a co-author [1,21–25,28], which can also be a product of power imbalance among project collaborators [22].

The cycle of credit that links publication records to all facets of scientific career development incentivizes these authorship irregularities. Researchers may award guest authorship to colleagues in hopes of receiving reciprocal authorship on that colleague’s publications or to increase the likelihood of an article’s publication due to that colleague’s political or reputational influence [27]. Omission of legitimate co-authors could benefit acknowledged authors by inflating the latter group’s perceived contributions to a study [25]. Such practices can help bolster a researcher’s publication record and by extension further his or her career by exaggerating expertise and accomplishments.

Authorship practices are well studied in health and biomedical sciences [27], and a recent review of this literature showed author contributions were often inconsistent with the ICMJE guidelines [29]. Many individual studies have documented the prevalence of authorship irregularities in the biomedical research field [30–33]. Approximately 10 to 20% of surveyed lead authors of medical research articles reported honorary authorship among their co-authors [31,33] while nearly one third of co-authors lacked the intellectual contributions needed to fulfill ICMJE requirements [30,32]. Ghost authorship is also prevalent in biomedical research with approximately 20 to 30% of surveyed authors identifying either direct involvement in the practice or awareness of a colleague wrongfully denied authorship [34,35]. These practices devalue authorship and diminish the sense of contribution and responsibility normally associated with the author designation [29].

While researchers have quantified author contributions to scientific manuscripts in a variety of publications for biomedical, health, and social science research [27], to our knowledge, only two studies [36,37] have examined this issue for the field of ecology. Modern ecological research is cross-disciplinary, and individual studies often integrate researchers from a variety of scientific fields [38–42]. Consequently, authorship practices in ecological research represent a broad range of individual scientific disciplines (e.g., social, political, economic, natural) and groups (e.g., governmental, academic, non-governmental organizations). The proportion of large, multi-authored studies in ecology [28,37], like other research fields [4,38,39,40,41,42,43,44,45,46,47,48], has increased in recent decades. This historical increase in the number of co-authors per study could be a result of a diverse array of changes in research and authorship practices so these trends make author contributions even less clear. Increases in individual specialization, multi-disciplinary research, international collaborations, and dilution of individual author contributions are all potential explanations for observed growth in the number of authors per study [41,45]. The previous publications on authorship issues in ecology [36,37] noted challenges in defining authorship in ecological publications due to such increases in both cross-disciplinary research and multi-authored studies. Based on issues raised in qualitative authorship surveys, the study authors proposed the adoption of a byline statement system defining co-author contributions for ecological research journals [36,37]. The proposed byline system has yet to be adopted by most ecological journals and so author contributions to ecological studies are still poorly defined. To better understand author contributions for recent publications in the field of ecology, we surveyed lead authors for papers published in 2010 regarding the contributions of co-authors on their respective publications.

## Methods

### Authorship survey design

To evaluate author contributions to current ecological publications, we sent surveys to lead authors of randomly selected articles in ecological journals. Human participants in this study consisted of anonymous respondents to an electronic survey. All participation was completely voluntary and data were collected in a manner that maintained anonymity of all participants. We randomly selected articles from journals listed in the 2009 Thomson Reuters’ ISI Web of Knowledge Science Citation Index with an impact factor ≥ one under the “Ecology” subject heading (n = 83 journals). For each journal, we randomly selected twelve primary research articles (n = 996) published in 2010 with multiple authors. In selecting potential articles for inclusion in our survey, we used a two-stage random number generator. For each journal article, we first determined the issue with a random number generator set to match the total number of issues published for that journal in 2010. We then selected the specific article for inclusion in the survey using a random number generator set to the number of research articles published in the selected issue in chronological order. For example, random numbers two and three would correspond to the third research article listed in the second issue of a given journal. We then sent the first author on each article an email requesting his or her participation in an anonymous survey about authorship in ecological studies with a link to the survey. We distributed email survey participation requests from November 2010 to January 2011. We asked participants to identify their occupations at the time that their articles were published and asked them to record the total number of co-authors listed on their articles. Participants then recorded their contributions as well as the contributions of each co-author. We provided a total of ten possible contribution categories, with the tenth category listed as “other” and including a space to enter in a description (Table 1). For each co-author, we asked lead authors to list all of the ten categories that applied. If the written “other” description was equivalent to one of the remaining nine categories, we assigned the appropriate number to it. We grouped remaining “other” entries that did not match the nine defined categories as their own tenth category (Table 2).

**Table 1:** List of potential authorship contributions provided in our author survey. If a co-author provided a service not captured in our nine listed categories, we instructed the survey participant to select “Other” and provide a brief written description of the contribution. Descriptions with superscripts represent contributions that match the Ecological Society of America (ESA)^1^ and International Committee of Medical Journal Editors (ICMJE)^2^ guidelines.

Author contribution
Conceived the ideas or experimental design of the study^1,2^
Performed experiments/data collection^1,2^
Data analysis and interpretation^1,2^
Primary author (wrote most of the paper or drafted the paper)^1,2^
Provided revisions to scientific content of manuscript^2^
Provided stylistic/grammatical revisions to manuscript
Provided funding
Provided access to crucial research components (field site, equipment, samples, data)
Principal investigator (advisor, head of project, manager)
Other

**Table 2:** List of “Other” contributions provided by co-authors in surveyed studies.

Number of Co-Authors in Study	Position of Author with “Other” Contribution	Occupation of Lead Author	“Other” Contribution
6	6	Professor	Deadbeat author
8	2–7	Conservationist	Contributed to conservation effort
13	7–12	Research Scientist	Part of group
5	3–4	Research Scientist	Nothing
3	3	Graduate Student	Laboratory technician, did not contribute to the research directly
12	11	Graduate Student	Local collaborator, necessary for research permit
6	5–6	Science Group Leader	Helped

### Survey participant group representativeness

We generated a sample-based rarefaction curve using the “iNEXT” package [43] in R [44] to evaluate whether our sample size of survey responses was sufficient to reflect the potential diversity of author contributions. In this approach, we adopted a method generally used in ecology to quantify species richness or diversity [43] and instead applied it to the estimation of the diversity of authorship contributions in our survey. We treated each unique combination of author contributions as a representation of the diversity of observed authorship contributions. For example, all authors in our survey that provided funding and also acted as principal investigators (PIs) were treated as a single authorship contribution group while all authors that provided funding, acted as PIs, and contributed to data collection were classified as a second unique group. We randomly sub-sampled increasing sample sizes of authorship survey responses (bootstrap set to 1,000) up to the actual survey sample size and further extrapolated estimates of the cumulative diversity of author contributions up to double the survey sample size. We plotted author contribution diversity estimates with respect to sample size. To assess the adequacy of our sample size, we compared the observed diversity of author contributions to the estimated diversity extrapolated based on a doubling of our actual sample size.

We also assessed whether the participant group differed from the total group of surveyed authors in terms of number of authors per study. Using a bootstrapping method, we selected a sub-sample of 449 studies 500 times and calculated median values for comparison with the median of the actual participant group. After testing for normality using a Shapiro-Wilk test and examining data Q-Q plots, we compared authorship number between studies with participating and non-participating lead authors using a Mann-Whitney U test due to significant departures from normality (P<0.001) and heavy tails in the data. We used both the “stats” and “coin” packages [45] in R to allow for the calculation of effect sizes.

### Author contributions by position

We performed hierarchical cluster analyses of author contributions for first, last, and middle authors separately for studies with > two co-authors to visualize the individual contributions that tended to co-occur among co-authors at those three positions. We used the “pvclust” package [46] in R for all cluster analyses. This package allows the user to assess uncertainty in hierarchical cluster analysis by generating approximately unbiased and bootstrap probability p-values for each cluster, which are computed by multiscale and normal bootstrap resampling, respectively.

### Author compliance with authorship guidelines

We calculated the percentage of all co-authors included in our survey that failed to meet ESA or ICMJE guidelines. Our survey did not explicitly ask about the last ICMJE requirement, approval of the final submitted version of the manuscript, so we were unable to evaluate this requirement. We further calculated the percent of co-authors that only provided a single contribution and/or did not contribute to the writing process (i.e., did not act as primary author or contribute stylistic or scientific edits). We calculated these percentages for both pooled results and for individual studies. We used pooled results across all surveyed studies to determine the total percentage of individual co-authors that fit a given contribution type. In a second calculation, we treated each surveyed study as an individual sampling unit. In this calculation, we determined percentages for each individual study, then averaged across studies to generate a mean and standard deviation for each contribution type. We included this latter approach to avoid potential bias from outlier studies with higher numbers of co-authors. We also calculated the percentage of all surveyed studies with at least a single co-author that possessed these same characteristics. We made these percent calculations separately for first (lead), last, and middle authors. Since middle authors were not included in studies with only two co-authors, we only made these separate calculations by author position for the subset of surveyed studies with > two co-authors (n = 341).

We performed logistic regression to assess the relationship between non-compliance with the ESA and ICMJE guidelines and the total number of co-authors per study as well as lead author occupation. We performed separate logistic regression analyses for a) all authors b) all lead authors, c) all last authors, and d) all middle authors. For the analyses that included all co-authors or all middle authors, we assigned a zero to each study only if all co-authors met the requirements for a given guideline and a one if ≥ one co-author failed to meet these guidelines. Since middle authors were not represented in studies with only two co-authors, we only performed separate logistic regression analyses on b), c), and d) for the sub-set of studies with > two co-authors. For each dataset, we performed the analysis for a) number of authors, b) lead author occupation, and c) both number of authors and lead author occupation. We then selected the model with the lowest Akaike information criterion corrected for small sample sizes (AIC_c_) value for each dataset using the equation:

$${AIC}_{c}=-2LogLikelihood+2K+\frac{2K(K+1)}{n-K-1}$$,

where K is the number of parameters and n is the sample size [47,48]. The first term reflects the deviance and tends to decrease with additional parameters while the second term penalizes model complexity by increasing with additional parameters. When combined, these two terms balance the tradeoff between bias and variance. In this approach, we converted individual model AIC_c_ values to ΔAIC_c_ values as Δ_*i*_ = AIC_ci_ − AIC_c min_ where AIC_min_ is the model with the lowest AIC_c_ value, and AIC_i_ is the AIC_c_ of a competing model with a higher AIC_c_ value. Larger Δi values reflect lower probability of model i being the best model of all candidate models considered in an analysis [47]. For logistic regression analyses we used the glm function with a binomial error structure and a logit link in the “MASS” package [49] in R. We calculated odds ratios and 95% confidence intervals for each analysis. We compared the proportions of survey respondents and guideline non-compliance by lead author occupation using the fisher.test function in the “fmsb” package [50] in R.

## Results

### Survey participant group characteristics

**Fig 1:**
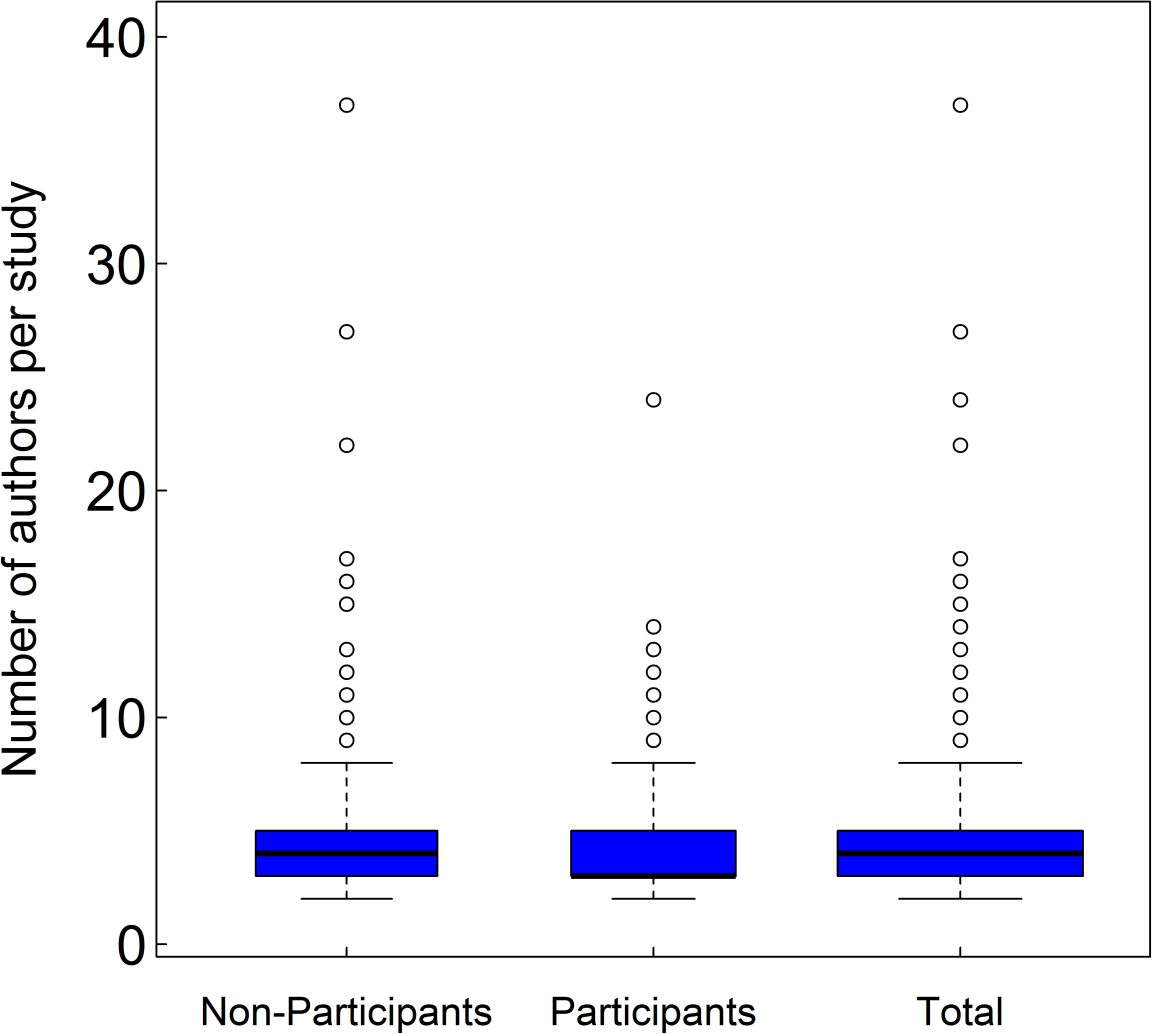
Box and whisker plots of the number of co-authors in manuscripts among survey participants, non-participants, and the total pool of selected studies. Black bars represent median values, blue boxes include the interquartile range (IQR), and “whiskers” reflect 1.5 IQR. Outlier values outside of this range are represented as individual points.

**Fig 2:**
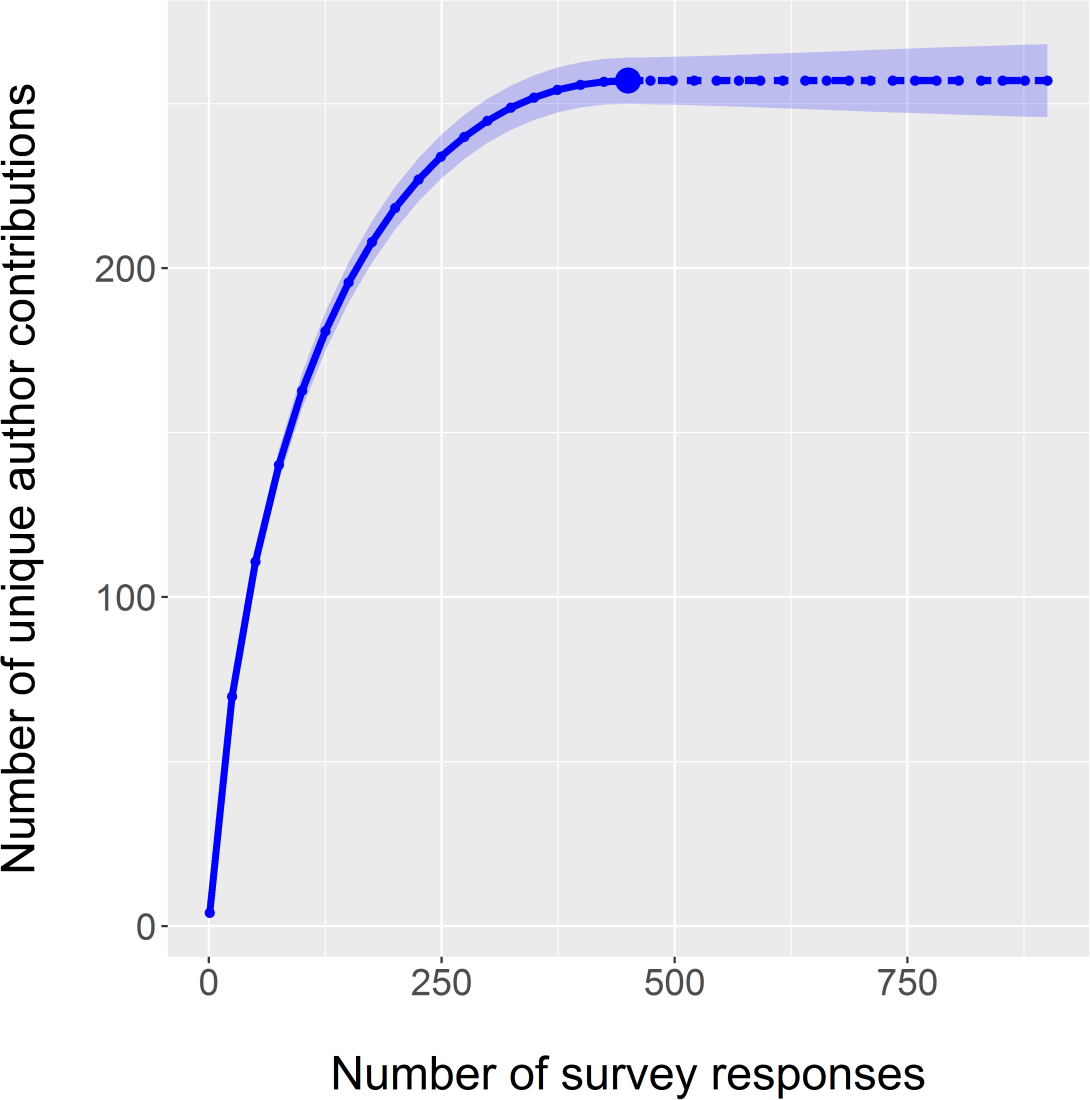
The cumulative diversity of individual author contributions among survey participants (n = 449). We generated estimates by randomly sampling increasing sample sizes of survey participants and calculating the total number of unique author contributions for each sample size. Shaded area reflects the 95% confidence intervals based on 1,000 repeated samples for each sample size. The solid blue line represents interpolated estimates, the solid blue circle is the observed diversity of contributions, and the dashed line is extrapolated estimates for sample sizes greater than our actual sample size.

We received complete responses from 45% of all surveyed lead authors. Survey participants were mostly graduate students (28%), post doctoral researchers (24%), research scientists (23%), and professors (17%) (Table 3). A bootstrapping analysis of 449 randomly selected survey responses produced a median of four co-authors per study 96% of the time and a median of three co-authors for the remaining 4% of replications. The median number of co-authors was three and four for the participant and non-participant groups, respectively (Fig 1). Mean ranks significantly differed between these two groups (Mann-Whitney U test: U = 112,210, z = 2.34, p = 0.019, r = 0.35). While the number of co-authors per study differed from the overall surveyed population, the effect size was relatively small [51]. The sample size of authors included in our analysis was sufficiently robust to capture the full diversity of author contributions as the observed diversity of contributions (n = 257) matched the extrapolated estimates even following a doubling of our actual sample size (Fig 2).

**Table 3:** Occupations of lead authors participating in our survey at the time that their manuscripts were submitted for review and associated study compliance rates with authorship guidelines. Percentages in a given column with different letter superscripts are significantly different (P<0.05) based on Fisher’s exact tests with Holm corrections for multiple comparisons.

Occupation	Participants (%)	Mean±SD Number of Co-Authors	ESA^1^ Non-Compliance (%)	ICMJE^2^ Non-Compliance (%)
Graduate Student	28^a^	4 ± 1.9	56 ^a^	80 ^a^
Post Doctoral Researcher	24 ^a,b^	4 ± 1.9	54 ^a^	76 ^a^
Research Scientist	24 ^a,b^	4 ± 2.4	41 ^a,b^	82 ^a^
Professor	17 ^b^	4 ± 3.0	22 ^b^	77 ^a^
Other	8 ^c^	5 ± 2.6	50 ^a,b^	70 ^a^

^1^Ecological Society of America

^2^International Committee of Medical Journal Editors

### Author contributions by position

**Fig 3:**
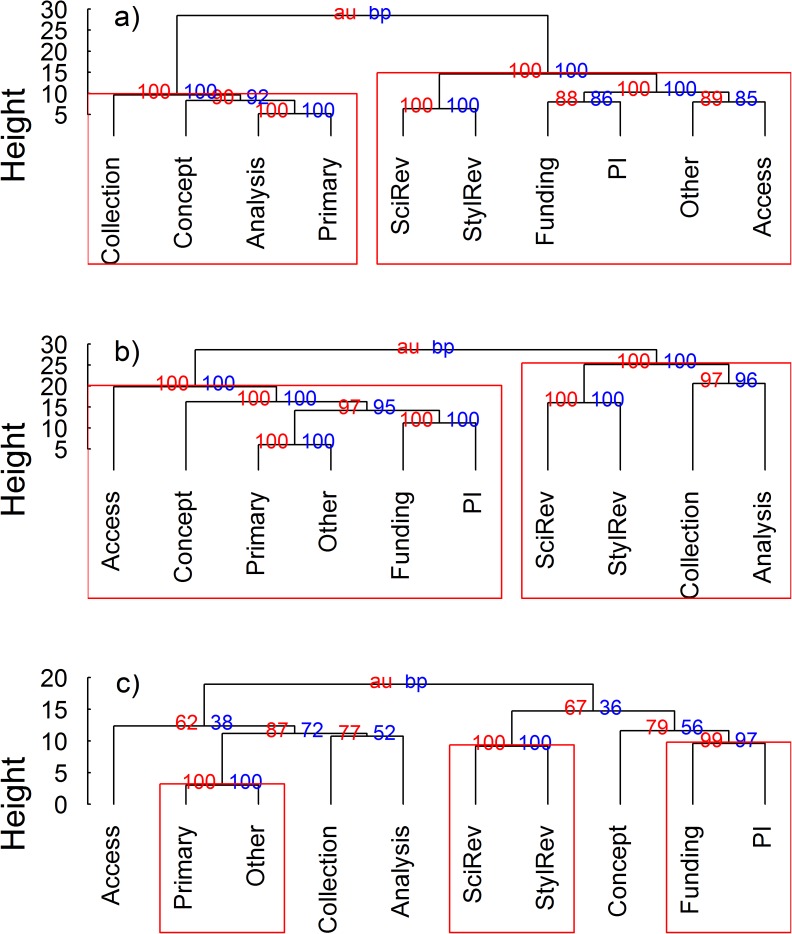
Hierarchical cluster analysis of author contributions for a) lead, b) middle, and c) last authors in surveyed studies with > two co-authors. Red values are approximately unbiased (AU) p-values, and blue values are bootstrap probability (BP) values (%). Clusters with AU values > 95% are enclosed in red boxes.

Author contributions varied by position. Most lead authors acted as the primary author while also contributing to developing the research concept and collecting and analyzing data (Fig 3). The majority (≥ 80%) of lead authors provided at least one of these contributions (Table 4). The small number of lead authors that lacked these contributions instead generally had an editorial role in the writing process and possessed attributes typical of a senior author (primary investigator, funder, source of resources for research). Significant pairing within the last author group included primary authors with other uncharacterized contributions, technical and stylistic editors, and a more honorary grouping of PI status and funder. Middle authors were similarly split. One group included a diverse array of contributions including attributes consistent with lead authorship like primary authorship and conception of the project idea. This group also included attributes consistent with senior authorship such as PI status and funding. A second group provided editing, data collection, and analysis, contributions more typical of a secondary author (Fig 3). About half of all middle and last authors provided technical edits. Middle and last author groups differed in several categories typical of differing author professional status. Nearly twice as many middle authors (46%) were directly involved in the actual data collection and experimental execution than last authors (27%). Funding and PI status contributions instead were more prevalent among last authors (Table 4).

**Table 4:** Percentage of co-authors that performed each survey contribution. To allow for direct comparison among author positions, calculations were made for the sub-set of studies with > two co-authors for lead, middle, and last authors (n = 341). For lead and last authors, contribution percentages are also reported in parentheses for the complete dataset (n = 449 studies).

Category	Lead	Middle	Last
Conceived ideas or experimental design^1^	85 (87)	22	32 (35)
Performed experiments/data collection^1^	80 (82)	46	27 (25)
Data analysis and interpretation^1^	93 (93)	37	28 (31)
Primary author^1^	97 (97)	2	2 (2)
Funding	25 (27)	15	35 (37)
Revisions to scientific content of manuscript	40 (42)	50	61 (62)
Stylistic/grammatical revisions	36 (38)	43	59 (61)
Other	0 (0)	2	1 (1)
Access	19 (22)	31	32 (33)
PI	31 (30)	13	43 (43)

^1^Signifies contributions that meet Ecological Society of America (ESA) authorship requirements. No single contributions meet International Committee of Medical Journal Editors (ICMJE) guidelines.

### Author compliance with authorship guidelines

Among all co-authors in the surveyed studies, 21% did not meet ESA guidelines while nearly half (47%) failed to meet the contributions outlined by the ICMJE (Table 5). Nearly half (48%) of all studies contained at least one co-author that failed to meet ESA guidelines, and most (78%) included a co-author failing to meet ICMJE guidelines. The higher percent of co-authors and studies failing to meet ICMJE guidelines was due to a high percentage of authors that did not participate in the writing process with 27% of all co-authors in 46% of all studies not contributing to writing (Table 5).

**Table 5:** Summary of author contributions for 449 manuscripts published in ecological journals in 2010.

Category	Percent of Authors (%)	Mean Percent per Study ± SD (%)	Percentage of Studies (%)
Did not meet ESA^1^ requirements	21	20 ± 24	48
Did not meet ICMJE^2^ requirements^3^	47	42 ± 28	78
Did not contribute to writing	27	21 ± 26	46
Only PI	1	1 ± 4	3
Only funding	<1	0 ± 3	1
Only PI and funding	1	0 ± 3	2
Only collected data^4^	7	4 ± 13	14
Only contributed to writing^4^	5	5 ± 13	16
Only conceived idea or design^4^	1	1 ± 6	2
Only analyzed data^4^	2	2 ± 8	7
Only primary author^4^	<1	0 ± 1	<1
Only edited paper	5	5 ± 12	15
Only provided access	3	2 ± 6	7
Only “Other”	1	1 ± 5	2

^1^Ecological Society of America

^2^International Committee of Medical Journal Editors

^3^Our survey did not explicitly ask about the last ICMJE requirement, approval of the final submitted version of the manuscript, so our classification of ICMJE compliance does not consider this third criterion.

^4^Signifies contributions that meet ESA authorship requirements. No single contributions meet ICMJE guidelines.

Authorship guideline compliance varied by author position. Among studies with > two co-authors, all lead authors met the ESA guidelines and only 2% failed to meet the ICMJE guidelines (Table 6). While non-compliance for ICMJE guidelines was similar for last and middle authors, the former group had a higher incidence of non-compliance for the ESA guidelines (Table 6). For ESA guidelines, last (37%) authors had a higher non-compliance than middle (24%) authors (Table 6). Middle (63%) and last (60%) authors had similarly high non-compliance rates with the ICMJE guidelines. Among last authors, 4% provided only funding and PI status while an additional 2% contributed only one of these two categories. Data collection was more prevalent as a sole contribution for middle (11%) than last (5%) authors and occurred in 14% of studies. Similar percentages of middle (7%) and last (7%) authors only contributed to the writing phase of a study. Approximately 15% of all studies included a co-author that functioned only as an editor or writer (Table 6).

**Table 6:** Summary of author contributions for 341 manuscripts published in ecological journals in 2010 with > two co-authors. The “All Authors” column shows the percentage of all authors represented in our survey with a given contribution category. The remaining three columns show the percentage of authors of a given position (lead, middle, or last) within the same contribution category. For middle authors, we report both pooled averages as well as mean ± SD in parentheses.

Category	All Authors (%)	Lead Authors (%)	Middle Authors (%)	Last Authors (%)
Did not meet ESA^1^ requirements	22	0	24 (23±35)	37
Did not meet ICMJE^2^ requirements^3^	49	2	63 (57±42)	60
Did not contribute to writing	29	2	39 (30±39)	27
Only PI	1	0	1 (1±5)	2
Only funding	<1	0	0 (0±1)	2
Only PI and funding	1	0	0 (0±6)	4
Only collected data^4^	7	0	11 (8±21)	5
Only contributed to writing^4^	6	<1	7 (7±20)	7
Only conceived idea or design^4^	1	<1	1 (1±7)	1
Only analyzed data^4^	2	0	3 (3±14)	2
Only primary author^4^	<1	<1	0 (0±0)	0
Only edited paper	6	0	7 (7±19)	7
Only provided access to research components	3	0	4 (2±9)	3
Only “Other”	1	0	2 (1±7)	1

^1^Ecological Society of America

^2^International Committee of Medical Journal Editors

^3^ Our survey did not explicitly ask about the last ICMJE requirement, approval of the final submitted version of the manuscript, so our classification of ICMJE compliance does not consider this third criterion.

^4^Signifies contributions that meet ESA authorship requirements. No single contributions meet ICMJE guidelines

**Fig 4:**
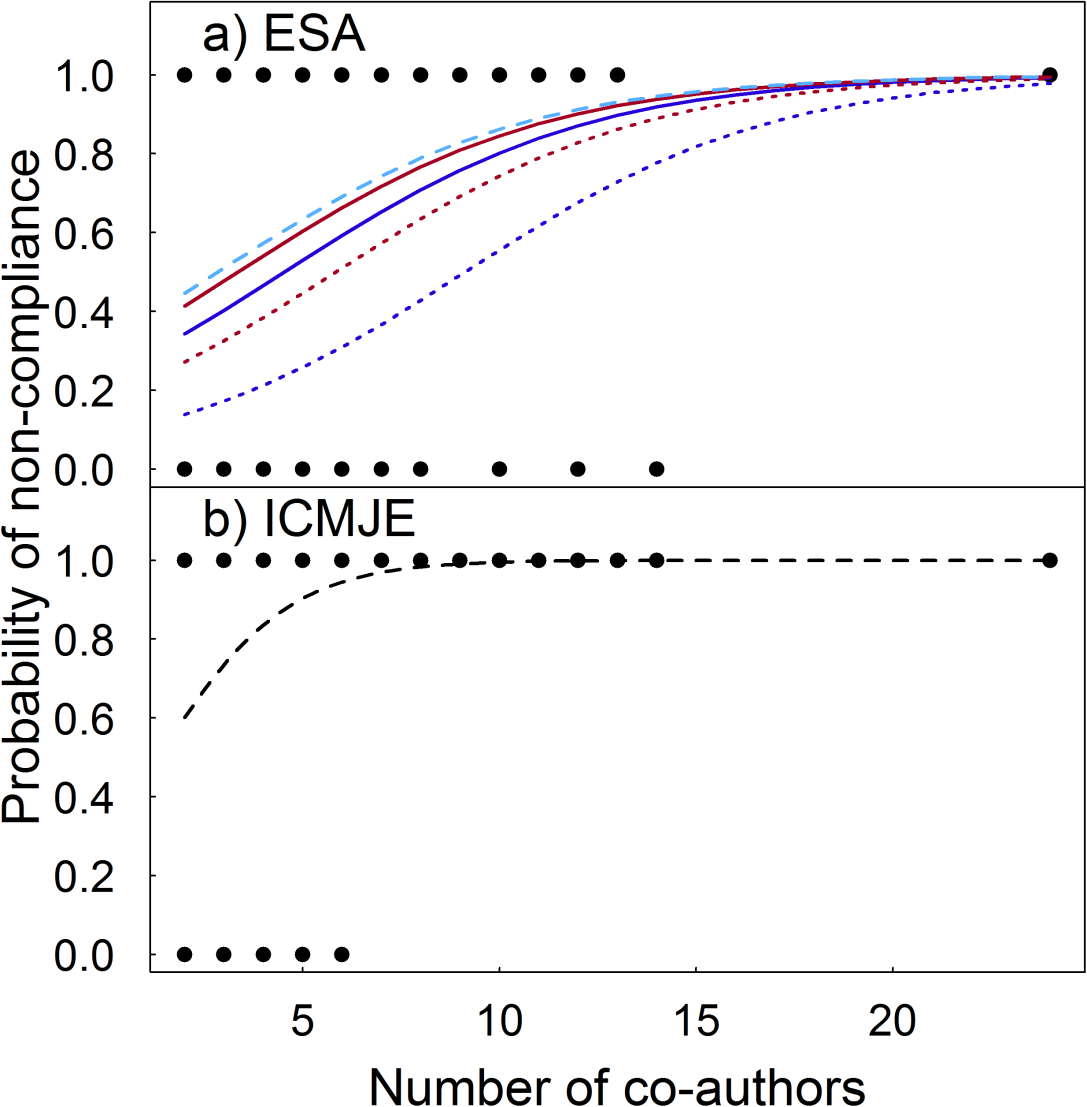
Plots showing the probability of study non-compliance with a) Ecological Society of America (ESA) and b) International Committee of Medical Journal Editors (ICMJE) guidelines in relation to the number of co-authors per study. For ESA guidelines, probability of non-compliance also varied in relation to lead author occupation. Separate curves reflect non-compliance probability for studies led by graduate students (dashed light blue), post doctoral researchers (solid red), other professions (solid dark blue), research scientists (dotted red), and professors (dotted light blue). Our survey did not explicitly ask about the last ICMJE requirement, approval of the final submitted version of the manuscript, so our classification of ICMJE compliance does not consider this third criterion.

The probability of a study containing an author that did not meet ESA or ICMJE guidelines increased with the number of co-authors, and lead author occupation also affected ESA compliance rates (Tables 7 and 8). For all co-authors, the odds of study non-compliance increased by 1.3 (ESA) and 1.8 (ICMJE) times for each additional co-author per study. The proportion of ESA non-compliance was higher for studies led by graduate students and post doctoral researchers relative to professor-led studies (Table 3; P<0.001 for both comparisons). In relation to professor-led publications, probability of ESA non-compliance was higher for studies led by all other occupations (Fig 4). Study non-compliance with ICMJE guidelines did not significantly vary among lead author occupations and was similarly high (≥70%) for all groups (Table 3). There was no significant relationship between the number of co-authors and failure to comply with either guideline set for lead or last authors although last authors in studies led by graduate students and post doctoral researchers were more likely to violate ESA guidelines than last authors in professor-led studies (Table 7). The probability of middle authors being non-compliant was significant with respect to the number of co-authors for ICMJE guidelines (Table 7). For ESA guidelines, the probability of non-compliance significantly varied with lead author position and the number of co-authors for middle authors (Table 7). Studies led by graduate students had a higher probability of non-compliance among middle authors than professor-led studies. All studies with ≥ seven co-authors contained at least one author that failed to meet ICMJE contribution requirements (Fig 4).

**Table 7:** Results of logistic regression analyses of the probability of Ecological Society of America (ESA) and International Committee of Medical Journal Editors (ICMJE)^1^ non-compliance in relation to the number of co-authors and lead author professional position. All author position data are scaled relative to professor lead authors.

	ESA					ICMJE
Dataset	Odds Ratio					Odds Ratio
	(95% CI)					(95% CI)
	P-value					P-value
*All studies*	Post Doctoral Researcher	Research Scientist	Graduate Student	Other	Number of Authors	Number of Authors
All co-authors	4.39	2.32	4.99	3.24	1.29	1.84
	(2.25–8.91)	(1.18–4.74)	(2.62–9.98)	(1.10–9.69)	(1.17–1.44)	(1.51–2.29)
	P<0.001^*^	P = 0.017^*^	P<0.001^*^	P = 0.033^*^	P<0.001^*^	P<0.001^*^
*Studies with > two co-authors*						
All co-authors	4.78	2.10	4.65	3.18	1.27	1.58
	(2.24–10.74)	(0.98–4.68)	(2.22–10.24)	(0.90–11.80)	(1.13–1.44)	(1.23–2.13)
	P<0.001^**^	P = 0.062	P<0.001^**^	P = 0.074	P<0.001^**^	P = 0.001^**^
Lead authors	^**^	^**^	^**^	^**^	^**^	0.92
						(0.56–1.21)
						0.681
Last authors	3.32	1.94	2.57	2.12	NA	1.03
	(1.55–7.56)	(0.89–4.47)	(1.22–5.77)	(0.56–7.54)		(0.94–1.15)
	P = 0.003^*^	P = 0.104	P = 0.017^*^	P = 0.249		P = 0.498
Middle authors	2.44	1.72	3.76	5.72	1.57	2.14
	(0.97–6.73)	(0.67–4.81)	(1.56–10.18)	(1.40–24.88)	(1.37–1.83)	(1.67–2.83)
	P = 0.069	P = 0.279	P = 0.005^*^	P = 0.016^*^	<0.001^*^	P<0.001^*^

^1^Our survey did not explicitly ask about the last ICMJE requirement, approval of the final submitted version of the manuscript, so our classification of ICMJE compliance does not consider this third criterion.

*Co-variate significant (P<0.05)

**No lead authors failed to meet ESA guidelines.

NA Data not presented because co-variate not included in best model (i.e., model with lowest AIC_c_ value).

**Table 8:** Model Akaike information criterion with correction for small sample sizes (AIC_c_) comparisons of probability of authorship non-compliance with the Ecological Society of America (ESA) and International Committee of Medical Journal Editors (ICMJE)^1^ authorship guidelines. Values are ΔAIC_c_ (AIC_c_ Model—AIC_c_ Minimum). The best model for each dataset has the lowest AIC_c_ value (i.e., ΔAIC_c_ = 0).

		ESA			ICMJE	
Dataset	Number of Co-Authors	Lead Author Position	Number of Co-Authors + Lead Author Position	Number of Co-Authors	Lead Author Position	Number of Co-Authors + Lead Author Position
*All studies*						
All co-authors	22.55	26.75	0.00	0.00	54.82	5.79
*Studies with > two co-authors*						
All co-authors	16.21	15.76	0.00	0.00	21.82	6.68
Lead authors	*	*	*	0.00	2.01	3.78
Last authors	4.33	0.00	1.62	0.00	2.43	4.13
Middle authors	4.61	50.72	0.00	0.00	61.89	5.33

^1^ Our survey did not explicitly ask about the last ICMJE requirement, approval of the final submitted version of the manuscript, so our classification of ICMJE compliance does not consider this third criterion.

*No lead authors failed to meet ESA guidelines.

## Discussion

Our survey results revealed a disconnect between guidelines proposed by the ESA and ICMJE and actual author contributions to ecological studies. All (ESA) or most (ICMJE) co-authors failing to meet these guidelines were secondary authors. Lead authors consistently met the requirements for both guidelines through involvement in the conception of the study, participation in data collection and analysis, and writing. Compliance was similar for medical research, where 99% of lead authors met ICMJE requirements [32]. This consistency transfers a clear meaning to the lead author position in ecological studies as almost all surveyed lead authors were involved in the major facets of the research and as such should be able to defend their studies. In addition to the larger subset of lead authors involved in all major research facets, our cluster analysis identified a second grouping among lead authors more typical of a senior author (i.e., editorial role combined with access, funding, and PI status). This second group provides evidence that the small percentages (2% in our survey) of lead authors failing to meet ICMJE contribution requirements are heads of labs rather than junior level researchers. Secondary authorship instead lacks clear meaning due to the diversity of contributions observed within this group in which roles ranged from “nothing” or “deadbeat author” (see Table 2) to comprehensive involvement equivalent to lead authors. This ambiguity dilutes authorship value for non-lead authors since readers cannot distinguish gift authors from authors that provided constructive contributions. Our cluster analysis revealed two general groups among middle authors, one characteristic of more senior level researchers (e.g., funding, PI status, conception of design) and the other more typical of junior level researchers (e.g., data collection and analysis) [52]. This dichotomy among middle authors further limits readers’ ability to evaluate co-author roles in published research. Secondary author compliance with ICMJE requirements for medical research studies varied from 85% to only 53%, depending on position [32]. Similar to findings for medical research [32], a high proportion of co-authors in our survey did not participate in the writing process and, in cases where singular ESA requirements were met, may have provided an important contribution to earlier phases of a research project. In instances where neither guideline was met, the co-author may have been a gift or guest author. In both cases, the co-author would be unable to provide input or approval towards the final synthesis and interpretation of a dataset. In the former case, any questions of authorship legitimacy could be resolved by involving that co-author in the editing process. Even in cases where a co-author had no involvement in the earlier phases of a study, she or he could provide intellectual contributions and be able to defend study results through critical editing of the scientific content during the writing phase.

We cannot assign causality to observed increasing trends [36,53] in co-author numbers per study in ecology without equivalent author contribution survey data from previous decades, but certain facets of our survey results do offer some insight into modern trends. Our study showed an increased probability of non-compliance as co-author numbers increase, suggesting that larger multi-authored studies are in fact contributing to authorship dilution. Studies of publications in other research fields have shown honorary [54–56] or “undeserved” [57] authors to be more prevalent in studies with more co-authors. Similarly, individual author contributions decreased with increasing number of co-authors per study in clinical and general science publications [30,32]. If non-compliant co-authors are randomly distributed across the overall population of co-authors in a given research field then larger studies would be more likely to contain a non-compliant co-author simply due to the fact that they represent a larger overall number of co-authors. Non-compliance in larger studies may be more prevalent due to an associated dilution of contact among co-authors in such studies. Large, multi-authored studies may include co-authors that lack direct contact with the lead author (e.g., collaborators of collaborators), which would limit the lead author in her or his ability to evaluate the legitimacy of contributions of all co-authors in a study.

Individual middle authors in our survey were less likely to meet ESA and ICMJE requirements in larger multi-authored studies while last author compliance did not vary as a function of the number of co-authors. Observed trends for middle authors may be a result of individual authors in such studies having more specialized, one-dimensional roles and/or a higher rate of gift authorship in larger studies. In a survey of author contributions, 30% (clinical research) and 21% (general science research) of secondary authors did not provide any intellectual contributions [30]. Assignment of last author positions varies among research disciplines and could correspond to authors with the smallest contributions or senior authors [9,58]. Author order can also be assigned alphabetically in some research fields (e.g., physics, economics, and information science [59–62]), although to our knowledge this practice is not typical in ecology. Our cluster analysis of authorship contributions showed smaller significant pairing groups among last authors consistent with multiple, distinct author contribution categories. Some last authors had principally editorial roles, which could indicate lowest contribution and/or senior author status. Senior or guest authorship appeared to be more prevalent for this group than for middle authors, as one of the significant last author clustering groups consisted only of funding and PI status. This finding is consistent with the prevalence of senior level researchers in last author positions in biology and natural resources science publications [63]. This fragmentation of author contributions may partly explain the lack of correlation between non-compliance of last authors and number of co-authors. If the final author position was reserved for a guest author, a last author in a study with only three authors would not be any more or less likely to meet guideline requirements than a guest author with ten or twenty co-authors. If this position was only reserved for the co-author with the lowest contribution to a study, this lowest proportion might be expected to decrease with increasing co-author number.

With increased specialization and collaboration, a growth in the number of co-authors per study is an expected outcome that does not necessarily result in diminished co-author contributions. For example, all individuals in a manuscript with fourteen co-authors in our survey met ESA requirements, suggesting that each provided at least one legitimate contribution to the study. The probability of a study containing a non-compliant author was high in our survey even for publications with only a small number of co-authors, so the strength of authorship contributions is not a simple function of the number of co-authors in a study. Dual authored papers in our survey had a 60% probability of including an author failing to meet ICMJE guidelines and a probability as high as 40% for ESA guidelines (Fig 4). Given the high compliance rates of lead authors, these probabilities mainly reflect the limited contributions of second authors on dual authored publications and demonstrate that authorship contributions are ambiguous even for studies with few co-authors.

Study compliance varied in relation to lead author position for ESA guidelines but was high and uniform across lead author occupations for ICMJE guidelines. The ICMJE guidelines are more restrictive and as a result non-compliant authors can have a variety of contributions including those more typical of both junior level (e.g., data collection or analysis) as well as senior level (e.g., funding, PI status) researchers. Failure to meet the single faceted ESA guidelines instead is more consistent with honorary authorship as it means that a co-author was not involved in any facet of experimental design, data collection, analysis, interpretation, or manuscript writing. The proportion of ESA non-compliance was lowest for professor-led studies, which reflects a tendency towards more stringent co-authorship standards by professors relative to other professional positions. The frequency of honorary authorship in medical research was also reported at a lower rate for studies with more senior ranking lead authors [31]. The probability of last authors not complying with ESA guidelines was significantly higher for graduate student and post doctoral researcher-led studies than professor-led studies, which is consistent with assignment of honorary authorship to last authors in studies led by more junior level researchers. This pattern is also consistent with professors being listed last as senior authors for contributions outside of those set forth by the ESA (e.g., providing funding or acting as a program head). We did not ask survey participants to explain the rationale behind co-author selection, and so the factors driving differential non-compliance rates among lead author occupations remain unclear. The higher standard among professor-led studies could be due to greater autonomy and less vulnerability to pressured authorship than lead authors with lower professional status [37]. Oberlander and Spencer (2006) [22] reviewed graduate student authorship issues and noted that graduate students have an imbalance of power in relation to their supervisors. Based on this power imbalance as well as general inexperience, graduate students are vulnerable to exploitation [22]. The higher probability of honorary authorship among junior level lead authors may not always be pressured but instead could also reflect active decisions to include co-authors with greater professional experience as a means of increasing the likelihood of manuscript publication [22]. Understanding of authorship requirements may also vary by professional experience [37] with more experienced researchers (e.g., professors) being more likely to have established criteria for co-authors.

Research scientist-led studies did not proportionally differ in ESA-non compliance relative to the other three predominant lead author professions in our survey although probability of study non-compliance was higher for this group than professor-led studies. Unlike the professor group, research scientists likely include a broader spectrum of seniority as this group could include PIs heading their own labs as well as staff of professors and other senior level researchers. Consequently, intermediate ESA non-compliance for this group is consistent with inclusion of a mix of both junior and senior level researchers.

Several facets of our study likely resulted in conservative estimates of non-compliance with authorship guidelines. The survey group had a lower median number of co-authors than the non-participants (Fig 1) which, given the observed increase in probability of non-compliance with number of co-authors, likely means that our dataset had higher compliance than the overall sampled population. Our survey did not include the ICMJE requirement of approval of the final submitted version of the manuscript, so some of the authors that we classified as being in compliance with ICMJE guidelines may have actually been non-compliant. A survey of medical research found universal final approval among co-authors in less than half of surveyed studies [64], which, given other similar trends for ecological and medical research authorship practices, suggests that final approval was probably not provided in many studies in our survey. However, this “final approval” requirement was the ICMJE requirement with lowest compliance and level of understanding in a survey of medical researchers. Consequently, this requirement is more of an administrative requirement, like a conflict of interest or copyright transfer, and some medical researchers have recommended its exclusion from the ICMJE guidelines [65].

Other aspects of the survey could have artificially inflated authorship guideline non-compliance. For example, lead authors that experienced authorship abuses (e.g., pressured authorship) might be more likely to respond to our survey than authors for which co-author contributions were generally legitimate. Our survey approach in which we collected data only from lead authors may have imparted both positive and negative biases on our estimates of authorship guideline compliance. Our study design may have produced higher estimates of non-compliance for secondary authors than if we had individually surveyed each co-author. Conversely, our approach may have inflated the contributions of lead authors since they were able to self-assess contributions [55,66]. In a survey of author contributions in the Croatian Medical Journal [55], corresponding authors identified ICMJE compliance in only 28% of non-corresponding co-authors, but this estimate increased to 41% when based upon non-corresponding author self-assessment [55]. Self-reporting also produced a higher percentage of ICMJE-compliant authors than survey responses from co-authors for authors of articles in the Dutch Journal of Medicine [66]. True author contributions may not be completely reflected in either survey design as self-assessment may carry a slight positive bias while assessment of co-author contributions may instead have a negative bias.

Similarly, the requirements put forth by the ICMJE are open to interpretation. Surveys in other research fields revealed differing opinions among researchers regarding the meaning and validity of the individual guidelines [5]. We also found that some contributions did not always unambiguously match a specific requirement and consequently, other researchers may have had different classifications for some author contributions with regard to ESA or ICMJE guidelines. These differences reflect broader challenges in accurately defining authorship roles. No matter how tightly defined, authorship will always be subject to interpretation.

A byline or credit system has been widely described and recommended as a solution to authorship problems [1,5,21,22,24,28,32,36,37,67–75] and may present the best means of individually defining author contributions and appropriately crediting authors with their actual contributions. Bylines would provide proper identification of authors that have contributed to all facets of a study, whether listed as first, middle, or last authors. Conversely, authors that only provided funding or supervision would be properly identified as having a more limited role.

Researchers have also proposed various systems to quantify and rank individual author contributions (See review in Abambres and Arab (2016)) [76]. Clement (2014) proposed an authorship matrix method based on a modification of the ICMJE guidelines. In this approach, authorship status and order are determined by individual contributions to ideas, work, writing, and stewardship [77]. These elements are then weighted and estimates of individual percent contributions to each category are used to quantify the net contribution and responsibility of each individual to the overall study. Tscharntke et al. (2007) proposed that authors define the method used to determine order rank (e.g., alphabetical order, descending contributions) in the manuscript acknowledgments section and that author contributions be weighted based on position rank [78]. Abambres and Arab (2016) [76] recommended that author order be based on descending levels of contribution with the lead author contributing most and taking overall responsibility for the study.

All of these approaches are subject to the biases of those classifying individual contributions, which could inflate or deflate contributions if based on self-assessment or co-author assessment, respectively. The format of the authorship disclosure form can also bias reported prevalence and frequency of honorary authorship [55,56]. Checklist-style forms that clearly identified contributions satisfying ICMJE requirements classified significantly lower percentages of authors as honorary authors and identified honorary authors in significantly fewer studies than forms with open-ended or category-based contributions in medical journals [55,56]. This variability among forms also indicates another potential bias in our study. In a survey of authors in the Croatian Medical Journal, a category-based disclosure form like the one used in our survey classified 63% of authors in 69% of articles as honorary authors [56]. These estimates were significantly greater than classifications from a checklist-style form that identified 19% of authors from 33% of articles as honorary. An open-ended form identified a slightly lower percent of authors as honorary (55%) than the category-based form but honorary authors were found in more (83%) of the surveyed articles [56]. The checklist-style disclosure forms that identify contributions meeting ICMJE requirements likely impart a positive bias on authors to provide responses that satisfy editorial requirements [56]. General category or open-ended formats are less prone to bias, so while our study likely would have produced different estimates of ICMJE non-compliance using different survey formats, we feel that our category style was less prone to bias than a checklist format and more quantifiable than an open-ended survey.

Byline and other authorship classification systems can provide data on individual author contributions that are currently lacking in traditional formats, but the efficacy of such systems may require adoption of additional structural and cultural policies [37]. In addition to a byline system, Elliot et al. (2017) recommended that research teams develop written authorship policies and lead authors clearly define expected authorship criteria, funding agencies encourage such policies by requiring them in grant proposals, teams develop authorship committees to act as arbiters in authorship policy disputes, and universities provide training to educate researchers on authorship issues. These recommendations followed a qualitative survey of authorship practices of interdisciplinary science teams, which revealed a prevalence of honorary authorship. Motivations for honorary authorship included lack of training and understanding of required contributions, a desire for conflict avoidance, and power imbalances [37]. Issues associated with an imbalance of power and related gift or ghost authorship practices would not be resolved with any of the above-mentioned approaches in the absence of the recommended authorship committee. For example, authorship abuses related to power differentials among co-authors [22] are also unlikely to be resolved solely through a byline or credit system. While authorship committees might be able to resolve individual authorship disputes between junior and senior level researchers, junior scientists would still remain vulnerable to later forms of retaliation (e.g., exclusion from future collaborations, refusal to provide references) that could allow unethical authorship practices to persist [22].

While our study focused on ecological journals, the authorship contribution issues that we explored are central to all research science, and researchers have put forth similar calls for reform across many disciplines [29,36,62]. Given the central role that publication records play in research [3], authorship issues identified in our survey and similar surveys performed in other fields [27] raise serious questions about what authorship actually means in modern research. Our results suggest a certain level of ubiquity to observed ambiguities in author contributions given the cross-disciplinary nature of ecological research. Continued ambiguity in actual authorship contributions could result in the devaluation of publication records as intellectual currency since practices of gift and guest authorship inflate records of non-contributing authors. Without individual descriptions of all co-author contributions, legitimate authors cannot be distinguished from honorary authors.

## Conclusions

Lead author contributions in recent ecological publications are consistent and robust while secondary authors instead show a high level of individual variability in contributions and a low rate of involvement in the writing process. Almost half of all surveyed studies included a co-author that did not meet ESA requirements for authorship and non-compliance with ICMJE requirements occurred in over three fourths of all studies. Non-compliance for each set of guidelines was more common in studies with more co-authors, suggesting that observed rising trends in the number of authors per study in ecological journals [36,53] may also reflect a general dilution in co-author contributions. While it may not necessarily strengthen author contributions [79], a byline system would provide better individual identification of co-author contributions. A byline system would benefit from complementary policies that better define authorship criteria, foster team building and communication among collaborators, educate researchers on authorship ethics issues, provide a framework for resolving authorship disputes, and protect individuals against power imbalances [37]. Such advances are essential for the restoration of authorship meaning and value given our survey results showing highly variable and often limited author contributions in modern ecological research.

## Supporting information

S1 File Individual author contributions for all manuscripts included in our authorship survey analysis. (XLSX)

S2 File Lead author occupations, number of co-authors per study, and study non-compliance with Ecological Society of America and International Committee of Medical Journal Editors guidelines. (XLSX)
