# Correction: Author contributions to ecological publications: What does it mean to be an author in modern ecological research?

**DOI:** 10.1371/journal.pone.0187321

**Published:** 2017-10-26

**Authors:** John M. Logan, Sarah B. Bean, Andrew E. Myers

There is an error in reference 27. The correct reference is: Marušić A, Bošnjak L, Jerončić A. A systematic review of research on the meaning, ethics and practices of authorship across scholarly disciplines. PLoS ONE. 2011;6: e23477. doi: 10.1371/journal.pone.0023477

There is an error in reference 52. The correct reference is: Haüssler C, Sauermann H. Credit where credit is due? The impact of project contributions and social factors on authorship and inventorship. Res Policy. 2013;42: 688–703.

The second sentence of the fourth paragraph in the Introduction section should have cited reference 52 instead of 27.

The correct sentence should read: Researchers may award guest authorship to colleagues in hopes of receiving reciprocal authorship on that colleague’s publications or to increase the likelihood of an article’s publication due to that colleague’s political or reputational influence [52].

The fourth sentence of the last paragraph in the Introduction section should have cited references 36, 53 instead of 28, 37. In the same sentence, the references 38,39,40,41,42,43,44,45,46,47,48 should be replaced with the following omitted references:

Drenth JPH. Multiple authorship: the contribution of senior authors. JAMA J Am Med Assoc. 1998;280: 219–221.

Regalado A. Multiauthor papers on the rise. Science. 1995;268: 25.

Modi P, Hassan A, Teng CJ, Chitwood Jr WR. “How many cardiac surgeons does it take to write a research article?”: seventy years of authorship proliferation and internalization in the cardiothoracic surgical literature. J Thorac Cardiovasc Surg. 2008;136: 4–6.

Onwude JL, Staines A, Lilford RJ. Multiple author trend worst in medicine. BMJ. 1993;306: 1345.

Borry P, Schotsmans P, Dierickx K. Author, contributor or just a signer? a quantitative analysis of authorship trends in the field of bioethics. Bioethics. 2006;20: 213–220.

Epstein RJ. Six authors in search of a citation: villains or victims of the Vancouver convention? BMJ. 1993;306: 765–767.

Engelder T. The coupling between devaluation of writing in scientific authorship and inflation of citation indices. GSA Today. 2007;17: 44–45. Available from: https://www.geosociety.org/gsatoday/archive/17/7/pdf/i1052-5173-17-7-44.pdf.

McDonald RJ, Neff KL, Rethlefsen ML, Kallmes DF. Effects of author contribution disclosures and numeric limitations on authorship trends. Mayo Clin Proc. 2010;85: 920–927.

Shaban S. Multiple authorship trends in prestigious journals from 1950 to 2005. Saudi Med J. 2007;28: 927–932.

King C. Multiauthor paper redux: a new peak at new peaks. Science Watch Nov-Dec. 2007; Available from: http://ip-science.thomsonreuters.com/m/pdfs/klnl/8428096/swmultiauthor.pdf.

Cozzarelli NR. Responsible authorship of papers in PNAS. Proc Natl Acad Sci. 2004;101: 10495.

The references given in the sixth sentence of the last paragraph in the Introduction section are incorrect. In this sentence, the references 41, 45 should be replaced with the following omitted references:

Epstein RJ. Six authors in search of a citation: villains or victims of the Vancouver convention? BMJ. 1993;306: 765–767.

McDonald RJ, Neff KL, Rethlefsen ML, Kallmes DF. Effects of author contribution disclosures and numeric limitations on authorship trends. Mayo Clin Proc. 2010;85: 920–927.

The tenth sentence of the first paragraph in the Discussion section should have cited reference 27 instead of 52.

The correct sentence should read: Our cluster analysis revealed two general groups among middle authors, one characteristic of more senior level researchers (e.g., funding, PI status, conception of design) and the other more typical of junior level researchers (e.g., data collection and analysis) [27].

The structure of all affected sentences in the original article remains the same.
